# Postnatal serum IGF-1 levels associate with brain volumes at term in extremely preterm infants

**DOI:** 10.1038/s41390-022-02134-4

**Published:** 2022-06-09

**Authors:** William Hellström, Lisa M. Hortensius, Chatarina Löfqvist, Gunnel Hellgren, Maria Luisa Tataranno, David Ley, Manon J.N.L. Benders, Ann Hellström, Isabella M. Björkman–Burtscher, Rolf A. Heckemann, Karin Sävman

**Affiliations:** 1 Department of Pediatrics, Institute of Clinical Sciences, Sahlgrenska Academy, University of Gothenburg, Gothenburg, Sweden; 2 Department of Neonatology, Wilhelmina Children’s Hospital, University Medical Center Utrecht, Utrecht University, Utrecht, The Netherlands; 3 University Medical Center Utrecht Brain Center, Utrecht University, Utrecht, The Netherlands; 4 Section for Ophthalmology, Department of Clinical Neuroscience, Institute of Neuroscience and Physiology, Sahlgrenska Academy, University of Gothenburg, Gothenburg, Sweden; 5 Institute of Health Care Science, Sahlgrenska Academy, University of Gothenburg, Gothenburg, Sweden; 6 Institute of Biomedicine, Sahlgrenska Academy, University of Gothenburg, Gothenburg, Sweden; 7 Department of Pediatrics, Institute of Clinical Sciences, Skåne University Hospital Lund, Lund, Skåne Sweden; 8 Department of Radiology, Institute of Clinical Sciences, Sahlgrenska Academy, University of Gothenburg and Sahlgrenska University Hospital, Gothenburg, Sweden; 9 Department of Medical Radiation Sciences, Clinical Sciences, Sahlgrenska Academy, University of Gothenburg, Gothenburg, Sweden; 10 Region Västra Götaland, Department of Neonatology, The Queen Silvia Children’s Hospital, Sahlgrenska University Hospital, Gothenburg, Sweden

## Abstract

**Background:**

Growth factors important for normal brain development are low in preterm infants. This study investigated the link between growth factors and preterm brain volumes at term.

**Material/methods:**

Infants born <28 weeks gestational age (GA) were included. Endogenous levels of insulin-like growth factor (IGF)−1, brain-derived growth factor, vascular endothelial growth factor, and platelet-derived growth factor (expressed as area under the curve [AUC] for serum samples from postnatal days 1, 7, 14, and 28) were utilized in a multivariable linear regression model. Brain volumes were determined by magnetic resonance imaging (MRI) at term equivalent age.

**Results:**

In total, 49 infants (median [range] GA 25.4 [22.9–27.9] weeks) were included following MRI segmentation quality assessment and AUC calculation. IGF-1 levels were independently positively associated with the total brain (*p* < 0.001, *β* = 0.90), white matter (*p* = 0.007, *β* = 0.33), cortical gray matter (*p* = 0.002, *β* = 0.43), deep gray matter (*p* = 0.008, *β* = 0.05), and cerebellar (*p* = 0.006, *β* = 0.08) volume adjusted for GA at birth and postmenstrual age at MRI. No associations were seen for other growth factors.

**Conclusions:**

Endogenous exposure to IGF-1 during the first 4 weeks of life was associated with total and regional brain volumes at term. Optimizing levels of IGF-1 might improve brain growth in extremely preterm infants.

**Impact:**

- High serum levels of insulin-like growth factor (IGF)-1 during the first month of life were independently associated with increased total brain volume, white matter, gray matter, and cerebellar volume at term equivalent age in extremely preterm infants.
- IGF-1 is a critical regulator of neurodevelopment and postnatal levels are low in preterm infants. The effects of IGF-1 levels on brain development in extremely preterm infants are not fully understood.
- Optimizing levels of IGF-1 may benefit early brain growth in extremely preterm infants. The effects of systemically administered IGF-1/IGFBP3 in extremely preterm infants are now being investigated in a randomized controlled trial (Clinicaltrials.gov: NCT03253263).

## Background

Infants born preterm are at risk of impaired brain growth and maturation even in the absence of macrostructural brain damage.^1–3^ The third trimester is the peak period for brain maturation and development with incipient myelination, a four-fold increase in cortical folding, and increased dendritic arborization alongside a drastic increase in total brain weight, from less than 90 g in gestational week 22–23 to 400 g at term equivalent age (TEA).^4^

Technical advancements in high–resolution magnetic resonance imaging (MRI) now enable precise determination of brain growth by implementing volumetric segmentation tools. In preterm infants, brain volumes are typically reduced at TEA and later in life compared to healthy term infants. Volume reduction is linked to life-long impairments, including neurosensory, cognitive, and behavioral deficits.^5–9^

The growth factor most commonly linked to brain growth and maturation is insulin-like growth factor (IGF)-1. IGF-1 is involved in cell growth, survival, proliferation, and migration with brain-specific effects on synapse formation, myelination, and plasticity.^10^ Low systemic concentrations of IGF-1 characterize the postnatal period following preterm birth compared to corresponding intrauterine levels.^10^ Low IGF-1 levels are linked to a poor neurodevelopmental outcome at 2 years of age^11^ and altered brain volumes in moderately preterm infants born before 31 weeks gestational age (GA).^12^ In addition, experimental studies show that IGF-1 treatment in neonatal brain injury models in rodents and sheep boosted proliferation, differentiation, and survival of the oligodendrocyte lineage and subsequent myelin production.^13–16^ However, the link between IGF-1 and brain volumes has not been explored in the most immature preterm infants.

Numerous studies link other growth factors, including brain-derived neurotrophic factor (BDNF), vascular endothelial growth factor (VEGF), and platelet-derived growth factor (PDGF), to processes crucial in early brain growth and maturation.^17–20^ Despite this, there are limited data on how postnatal endogenous exposure to growth factors affect brain development in preterm infants.

This study investigates associations between postnatal endogenous exposure to growth factors and brain volumes at TEA in extremely preterm infants. We also relate our findings to GA at birth to study how immaturity affects the relation between growth factors and brain volumes.

## Methods

### Study population

**Fig. 1:**
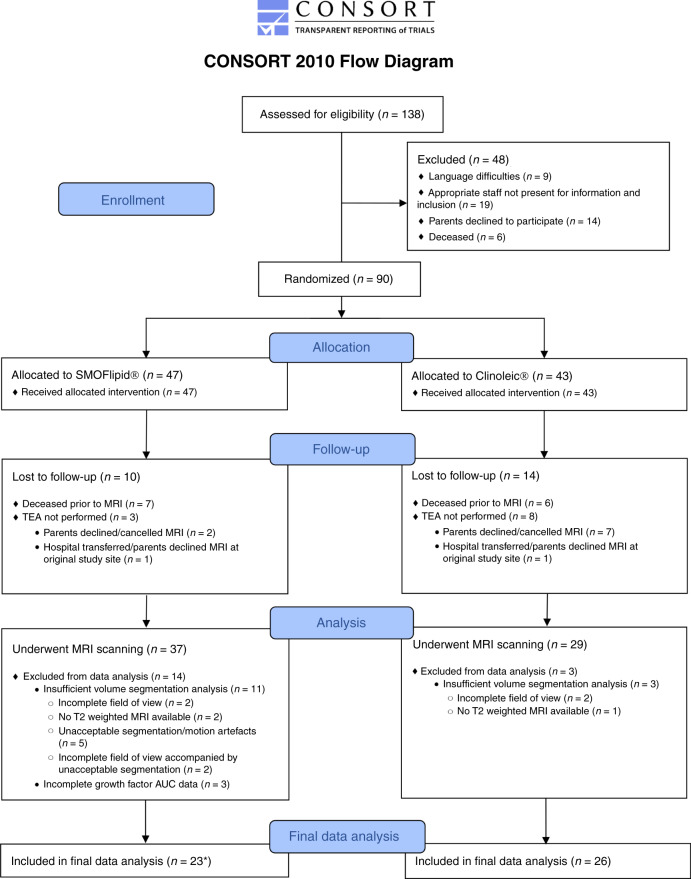
Consort study flowchart. In total, 49 infants were eligible for analysis with complete MRI volume segmentation and growth factor AUC. *In two infants, IGF-1 AUC was available, but not BDNF, VEGF, and PDGF. RCT randomized controlled trial, n number, TEA term equivalent age, IGF Insulin-like growth factor, BDNF brain-derived neurotrophic factor, AUC area under the curve, MRI magnetic resonance imaging.

The study included infants born <28 weeks GA, at Sahlgrenska University Hospital, Gothenburg, Sweden from 2013 to 2015 as part of a randomized clinical study investigating the effects of parenteral lipid emulsions on infant morbidities (Clinical trial NCT 02760472). The complete study protocol is available online.^21,22^ The inclusion flowchart is presented in Fig. 1. Pregnancies were dated by ultrasound at gestational week 17–18. The study was approved by the Regional Ethical Board, Gothenburg (Dnr 303–11; Clinical trial NCT 02760472), and infants were enrolled following written informed parental consent.

### Data collection and laboratory analysis

Serum aliquots were stored in a freezer at −80 °C until assayed. Blood was drawn from an umbilical or peripheral arterial catheter or venous puncture. For IGF-1 analysis, samples were diluted 1:50, and the IGF-1 concentrations were analyzed using a radioimmunoassay (Mediagnost GmbH, Tubingen, Germany), as described previously.^23^ For IGF-1, the intraassay coefficients of variation at concentrations of 9, 33, and 179 ng/l were 18, 9, and 7%, respectively, and the interassay coefficients of variation at concentrations of 9, 34, and 194 ng/l were 29, 11, and 8%, respectively, also described previously.^24^ BDNF, VEGF, and PDGF were analyzed using the ELLA multi-analyte platform (Bio-Techne, Minneapolis, MN), according to the manufacturer-provided protocol, and has been described in detail previously.^25^ In short, samples were diluted 1:4, and the interassay coefficients of variation for BDNF were at concentrations of 220, 7402, and 10,271 pg/ml, 7, 7, and 5%, respectively; for VEGF at concentrations of 35, 475, and 1667 pg/ml, 7, 7, and 8%, respectively, and for PDGF at concentrations of 7.5, 332, and 945 pg/ml, 5, 4, and 5%, respectively. Intraassay coefficients of variations for BDNF at 7731 pg/ml 6%, for VEGF at 502 pg/ml were 5%, and for PDGF at 983 pg/ml, 5%.

### MRI acquisition and evaluation

MRI scanning was performed at TEA on a 3 Tesla system (750W, GE Medical Systems, Waukesha, WI) using a 19- or 32-channel head coil. The scanning protocol matched the clinical routine and included 3D T1–weighted, T1 FLAIR, axial 2D T2–weighted (T2w), 3D T2w fast spin–echo, 3D susceptibility–weighted, and diffusion-weighted imaging. Only T2w images were used for volumetry. Acquisition parameters for 2D T2w were slice thickness 3 mm, repetition time 9278 ms, and echo time 74.5 ms. The 3D T2w were acquired with echo time 81–125 ms, slice thickness 0.8 mm, and repetition time 2740–3000 ms.

Segmentation analysis utilized T2w images. Using the automatic anatomical image segmentation described by Makropoulos et al.,^26^ volumes were determined for a set of brain regions. An atlas database consisting of expertly segmented reference images^27^ was applied with the DrawEM (Developing brain Region Annotation With Expectation–Maximization) module of the Medical Image Registration Toolkit.^26,28^

For segmentation analysis, a 3D image volume was built from each acquisition (2D and direct 3D), referred to as the image stack in the following. An experienced imaging scientist (R.A.H.) reviewed the segmentations and assigned a quality score, following a custom protocol. Image stacks showing quality deficiencies, making volume calculations unreliable, were excluded. If two or more image stacks of sufficient quality were available for the same infant, the best segmentation was selected for further analysis. Merged volumes of brain regions (total brain [i.e., total intracranial volume without cerebrospinal fluid], white matter, cortical gray matter, deep gray matter, and cerebellum) were generated by summation of selected individual regions.

Oral chloral hydrate (35 mg/kg) was used for sedation. A combination of purpose-made in-ear and over-ear sound absorption devices was used for hearing protection. All infants were closely monitored by a trained nurse or physician, including respiratory rate, oxygen saturation, and heart rate throughout the whole procedure.

### Statistical analysis and variable definition

Data were analyzed using IBM SPSS 26 (IBM, Armonk, NY). AUC values were retrieved using a trapezoidal method based on serum levels of growth factors from postnatal days (PND) 1, 7, 14, and 28. Both PND 1 and PND 28 values were needed for inclusion in data analysis in the AUC calculations. A *p* value <0.2 was required in the univariate analysis for inclusion in the final multivariable analysis. Independent variables included in the initial univariate analysis were postmenstrual age at the time of MR scanning (weeks), GA at birth (weeks), development in birth weight standard deviation score from birth until the time of MR scanning, total parenteral and enteral energy intake PND 1–28 (kcal), antenatal steroid treatment, small for gestational age (SGA), sepsis, significant brain injury, and sex. The model utilized was run on total brain volume, following model validation in subregions for eligibility. Included variables in the regression models were checked for multicollinearity (variance inflation factor >1, <2, and eigenvalue, accompanied by visual analysis), normal distribution of residuals, independent observations, and homoscedasticity. The variable significant brain injury was not included in the final model due to violation of multicollinearity diagnostics, in spite of an acceptable VIF score. This was accounted for by both a rerun of the analyzes and the variable significant brain injury and exclusion of infants with severe brain injury. Randomization to treatment was accounted for in multiple linear regression and did not have an association with brain volumes.

The threshold for the effect of GA on estimated probabilities of total brain volumes and IGF-1 used in the explorative subanalysis was identified using visual analysis of nominal data (weeks). IGF-1_high_ and IGF-1_low_ were defined as AUC values above and below the median, respectively. Development of weight SDS was defined as the difference between weight SDS at the time of MRI and weight SDS at birth.

The Spearman rank test was used for correlations between non-parametric data. For comparisons of non-parametric variables between groups and for categorical variables, the Mann–Whitney *U* test, *χ*^2^ test of independence, or Fisher’s exact test were used as appropriate. In analyzes, *p* values <0.05 were considered significant. To adjust for multiple hypothesis testing, the Holm–Bonferroni method was used.^29^ *P* values ≥0.01 are presented with two decimals, *p* values in the range 0.001–<0.01 are denoted with three decimals.

Clinical data were collected prospectively, and clinical diagnoses are listed in Table 1. MR-based macrostructural regions used were total brain volume (not including ventricles) and regional volumes (white matter, cortical gray matter, deep gray matter, and cerebellum). Significant brain injury was defined as intraventricular hemorrhage (IVH) grade III (according to Papile et al.^30^) and periventricular hemorrhagic infarction, white matter lesions (focal signal abnormality score ≥2 according to Kidokoro et al.^31^), cerebellar hemorrhage (signal abnormality score ≥2 according to Kidokoro et al.^31^), and/or cystic lesions (cystic lesion score ≥3 according to Kidokoro et al.^31^). TEA MR scanning was performed between postmenstrual age 39.7 and 49.9 weeks.

**Table 1:** Clinical characteristics, *n* = 49.

Gestational age, median (range) weeks	25.4 (22.9–27.9)
Birth weight, median (range) g	760 (455–1255)
Birth weight SDS^a^, median (range)	–0.7 (–4.1–1.3)
Total energy intake, median (range) kcal/kg/day	108.5 (94.0–145.4)
Gender male, number (%)	26 (53.1)
Sepsis^b^, number (%)	19 (38.8)
NEC^c^, number (%)	3 (6.1)
Any IVH^d^ or PVHI, number (%)	17 (35.4)
IVH Grade I–II, number (%)	14 (28.5)
IVH Grade III, number (%)	1 (2.0)
PVHI, number (%)	2 (4.1)
BPD^e^, number (%)	27 (55.1)
Any ROP^f^, number (%)	40 (81.6)
Postmenstrual age at MR scanning, median (range) weeks	42.8 (39.7–49.9)

*n* number, *SDS* standard deviation score, *NEC* necrotizing enterocolitis, *IVH* intraventricular hemorrhage, *PVHI* periventricular hemorrhagic infarction, *BPD* bronchopulmonary dysplasia, *ROP* retinopathy of prematurity, *MR* magnetic resonance.

^a^Birth weight SDS were computed according to ref. ^66^

^b^Sepsis was diagnosed by positive blood culture, except for *Staphylococcus epidermidis* where a concomitant CRP >20 mg/L was needed for diagnosis.

^c^NEC was diagnosed by clinical signs and radiological findings (Bell’s stages 2–3).

^d^IVH was diagnosed by repeated ultrasound examination and graded according to the Papile classification.^30^

^e^BPD was defined as the need for oxygen supplementation at 36 weeks postmenstrual age.

^f^ROP was classified according to the International Classification of Retinopathy of Prematurity.^67^

## Results

In total, 49 infants fulfilled growth factor AUC availability and MRI segmentation image quality criteria, Fig. 1. The clinical characteristics of the included infants are given in Table 1. Infants included in the final data analysis had similar characteristics as infants that did not meet MRI segmentation and AUC availability criteria, Supplementary Table 1.

**Fig. 2:**
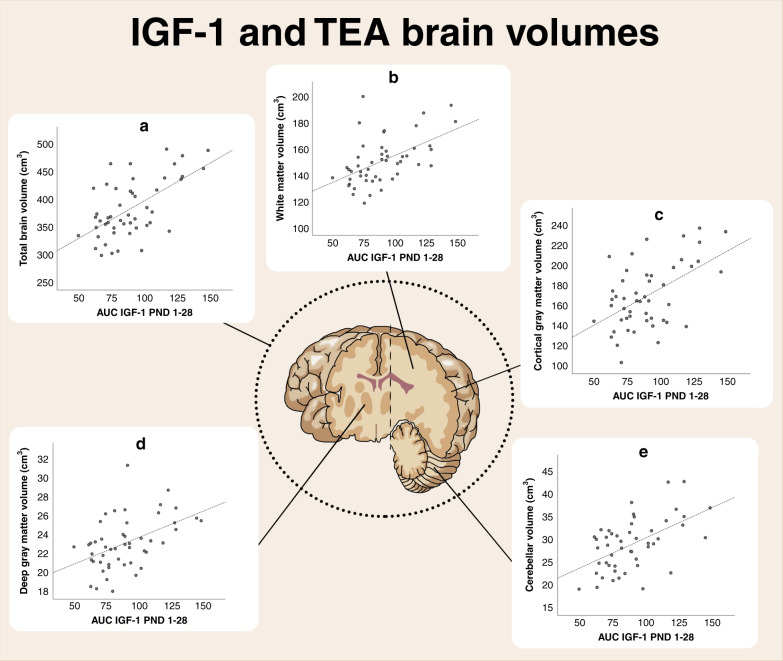
Serum levels of IGF-1 AUC (PND 1–28) and regional brain volumes at TEA. Higher IGF-1 levels were associated with total brain volume; *r* = 0.49, *p* < 0.001 (**a**), white matter volume; *r* = 0.55, *p* < 0.001 (**b**), cortical gray matter volume; *r* = 0.40, *p* = 0.004 (**c**), deep gray matter volume *r* = 0.45, *p* = 0.001 (**d**), and cerebellar volume; *r* = 0.47, *p* < 0.001 (**e**). IGF insulin-like growth factor, AUC area under the curve, PND postnatal day, TEA term equivalent age.

Larger IGF-1 AUC correlated with increased total brain volume, white matter volume, cortical gray matter volume, deep gray matter volume, and cerebellar volume in univariate correlation analysis, adjusted for multiple testing, Fig. 2a–e. Variables included in the initial univariate data analysis rendering the final statistical model are shown in Supplementary Table 2.

In the full statistical model adjusting for PMA at time of MRI (weeks) and GA at birth (weeks), higher serum levels of IGF-1 expressed as AUC were independently associated with total brain volume (*p* < 0.001, *β* = 0.90, 95% CI 0.41–1.39, *R*^2^ = 0.64), white matter volume (*p* = 0.007, *β* = 0.33, 95% CI 0.09–0.56, *R*^2^ = 0.30), cortical gray matter volume (*p* = 0.002, *β* = 0.43, 95% CI 0.17–0.70, *R*^2^ = 0.72), deep gray matter volume (*p* = 0.008, *β* = 0.05, 95% CI 0.01–0.08, *R*^2^ = 0.41), and cerebellar volume (*p* = 0.006, *β* = 0.08, 95% CI 0.02–0.13, *R*^2^ = 0.63), all remaining significant after adjusting for multiple testing. The presence of major cerebral injuries could not explain the associations as results were not affected by the inclusion of significant brain injury as a covariate in the full model or by excluding infants with significant brain injury (*n* = 18) (data not shown).

Brain volumes in relation to IGF-1_high_ and IGF-1_low,_ defined as above and below IGF-1 AUC median, are demonstrated in Table 2. The relationship between IGF-1 AUC and 84 anatomical subregions is shown in Supplementary Table 3. IGF-1 AUC positively correlated with volume (unadjusted) in 54 subregions of the brain, and ten subregions remained significant following adjustment for multiple testing.

**Table 2:** Absolute brain volumes (cm^3^) in relation to high (above median) and low (below median) IGF-1 AUC during the first 4 postnatal weeks of life.

	Total brain volume		White matter volume		Cortical gray matter volume		Deep gray matter volume		Cerebellar volume	
	Median	Min–max	Median	Min–max	Median	Min–max	Median	Min–max	Median	Min–max
Total, *n* = 49	366.4	297.5–489.1	149.0	118.6–199.6	164.2	102.9–236.3	22.9	18.0–31.3	29.1	19.0–42.6
IGF-1_low_, *n* = 25	356.6	297.5–462.8	139.3	118.6–199.6	159.5	102.9–210.8	22.0	18.0–26.5	26.5	19.0–32.0
IGF-1_high_, *n* = 24	411.4	306.3–489.1	155.2	128.4–192.9	186.4	122.7–236.3	23.7	19.7–26.5	31.0	19.1–42.6

*IGF* insulin-like growth factor, *AUC* area under the curve, *n* number.

BDNF AUC correlated positively with total brain volume (*r* = 0.34, *p* = 0.02), white matter (*r* = 0.40, *p* = 0.006), deep gray matter (*r* = 0.31, *p* = 0.03), and cerebellar volumes (*r* = 0.40, *p* = 0.005). No associations remained after adjusting for multiple testing or when adjusting for GA at birth and PMA at the time of MRI in the regression model. No associations were found for PDGF or VEGF and brain volumes at TEA.

No correlations were found between IGF-1 serum levels and relative brain region volumes (percentage of total brain volume, adjusted for intracranial volume), Supplementary Fig. 1 a–d. When comparing the relative brain region volumes between the IGF-1_high_ and the IGF-1_low_ group, the volume fraction of the cerebellum was proportionally higher in the IGF-1_high_ group than in the IGF-1_low_ group (*p* = 0.02), but the difference did not remain after adjusting for multiple testing, Supplementary Table 4.

**Fig. 3:**
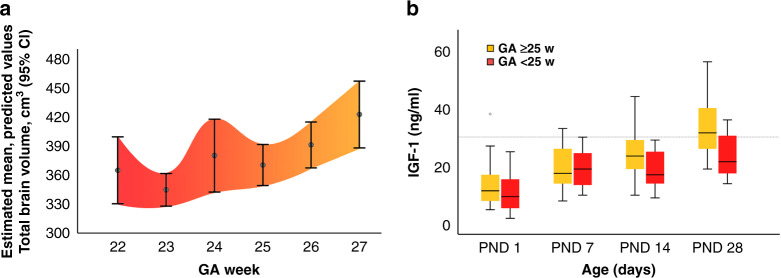
The impact of gestational age at birth on brain volume and serum IGF-1 levels. **a** Estimated unstandardized probabilities of total brain volume, retrieved from the full statistical model, illustrated per gestational week. Error bars indicate 95% CI. Colored area: interpolated. **b** Distribution of serum levels of IGF-1 during postnatal days 1, 7, 14, and 28 in relation to gestational age at birth (below or over 25 weeks). More immature infants show less increase in endogenous IGF-1 levels. Dotted line shows 30 ng/ml. A prolonged period with serum IGF-1 levels below this threshold has been related to morbidities in the neurovascular unit.^54^ Boxes illustrate interquartile range, whiskers show full range. IGF-1 insulin-like growth factor 1, CI confidence interval, PND postnatal day, GA gestational age.

We exploratively analyzed the role of GA on the link between IGF-1 and brain volumes. This was done by investigating the impact of IGF-1 AUC in the full statistical model adjusting for GA at birth and postmenstrual age at the time of MR scanning on the estimated unstandardized probabilities of total brain volume, illustrated per gestational week in Fig. 3a. Infants born at <25 weeks GA (*n* = 16) differed in their relationship between IGF-1 AUC and brain volumes when compared to infants born at ≥25 weeks GA (*n* = 33). Therefore, a threshold of GA of 25 weeks was used in the subanalysis. Infants born at <25 weeks GA had persistently low endogenous IGF-1 serum levels and did not show the increase in serum levels of IGF-1 over time seen in infants born at ≥25 weeks GA, Fig. 3a, b. In a subanalysis of infants born at <25 weeks GA, there was no association between IGF-1 serum levels and brain volumes. In contrast, higher IGF-1 levels corresponded to the larger total brain and larger white matter, cortical gray matter, deep gray matter, and cerebellar volumes in infants born ≥25 weeks GA. Similar results were seen when dichotomizing at median GA (25.4 weeks), Table 3. The postmenstrual age at the time of MR scanning was not significantly different in the infants born at <25 and ≥25 weeks GA, median (minimum–maximum) 43 (40–46.6) and 42.9 (39.7–49.9) weeks, respectively.

**Table 3:** Correlation of absolute brain volumes (cm^3^) and correlation to IGF-1 AUC stratified by gestational age at birth.

	Total brain volume		White matter volume		Cortical gray matter volume		Deep gray matter volume		Cerebellar volume	
	*r*_Spearman_	*p* value	*r*_Spearman_	*p* value	*r*_Spearman_	*p* value	*r*_Spearman_	*p* value	*r*_Spearman_	*p* value
GA <25 w, *n* = 16	0.03	0.93	0.23	0.39	0.03	0.91	–0.22	0.41	–0.06	0.82
GA ≥25 w, *n* = 33	0.58**	<0.001	0.57**	<0.001	0.55**	<0.001	0.57**	<0.001	0.59**	<0.001
GA <median^a^, *n* = 23	0.15	0.51	0.22	0.30	0.12	0.59	–0.09	0.70	0.01	0.96
GA ≥median^a^, *n* = 26	0.55**	0.003	0.45*	0.02	0.54**	0.004	0.56**	0.003	0.55**	0.004

*IGF* insulin-like growth factor, *AUC* area under the curve, *GA* gestational age, *n* number, *w* week.

^a^Median at 25.4 weeks.

*p < 0.05; **p < 0.01.

## Discussion

In this study of extremely preterm infants, we show that increasing IGF-1 serum levels are independently associated with increased total brain volume, as well as increased regional white matter, cortical gray matter, deep gray matter, and cerebellar volumes at TEA. At the same time, no associations were found for PDGF, BDNF, or VEGF.

Expression of IGF-1 in the brain is found in the cortex, cerebellum, hypothalamus, hippocampus, and spinal cord,^32^ with a peak in the perinatal period and a decrease when neuronal proliferation ceases.^33^ IGF-1 plays a crucial regulatory role for early neuronal maturational and differentiation processes, mainly via the PI3-K–Akt pathway.^34,35^ It affects vital neurodevelopmental processes such as the development of astrocytes and oligodendrocytes, synapse formation, myelination, and production of neurotransmitters.^36^ The main proportion of circulating IGF-1 is synthesized in the liver, but IGF-1 is secreted by almost all fetal tissues at some developmental stage and exerts its actions in endocrine, paracrine, and autocrine manners by binding to the IGF-1 receptor.^37^ In addition to endogenous brain expression, IGF-1 is also actively transported via the choroid plexus from the circulation into the nervous system and acts together with brain-derived IGF-1.^38,39^ Systemic levels of circulating IGF-1 may thus reflect IGF-1-mediated actions within the nervous system, as suggested by the findings in our study.^32^

The effect of IGF-1 on oligodendrocytes has been demonstrated in several studies. Our findings harmonize with a study of more mature infants, <31 weeks GA at birth, and with less pronounced depression of serum IGF-1. Pupp et al.^12^ observed an association between IGF-1 levels and total brain, unmyelinated white matter, and cerebellar volumes using a lower anatomical resolution MRI method. The association was restricted to cerebellar volume when adjusting for SGA.^12^ Carson et al.^40^ observed that overexpression of IGF-1 in a mouse model was associated with a 55% increase in brain size and an increase in myelin content by 130%, suggesting a specific effect of IGF-1 on white matter development.^40^ Following these findings, an increased percentage of myelinated axons and increased thickness of the myelin sheath have been found following increased expression of IGF-1 in transgenic mice.^41^ Similarly, in a preterm rabbit pup model, low IGF-1 levels were linked to decreased cerebellar external granular layer proliferation and decreased Purkinje cell maturation.^42^ IGF-1 thus seems to be of specific importance from a preterm clinical perspective, as early white matter abnormalities, as well as cerebellar hypoplasia, have been linked to later neurodevelopmental impairments.^43,44^ More specifically, white matter abnormalities at TEA have been associated with neurosensory impairments, cognitive delay, motor delay, and cerebral palsy at 2 years of age and motor impairment, cognitive impairments, special assistance requirements at school, and cerebral palsy later in childhood.^43,45,46^ Early quantitative measurements of the cerebellum have been associated with motor behavior during the first 2 years of life, as well as cognitive development; however, further studies are required to elucidate the exact role of a reduced size of the cerebellum.^47–50^

To explore the association between IGF-1 and brain regions of specific functional importance in the preterm infant, we investigated the association between IGF-1 and the relative volume (percentage adjusted for intracranial volume) of particular brain regions. No clear associations were found, and the tendency towards a larger relative volume of the cerebellum in infants with high levels of systemic IGF-1 did not remain significant after correction for multiple comparisons. This suggests that systemic IGF-1 levels are related to global brain growth rather than the growth and maturation of specific regions. However, this does not exclude that the reduced volume may be of particular importance in areas such as white matter or cerebellum that are commonly linked to adverse outcomes in the preterm infant.

The third trimester is a critical period in brain development, encompassing myelination, synaptogenesis, and neuronal organization, alongside the development of functional capacity. Preterm birth results in morphological brain alterations, including reduced brain growth, that persist until adulthood even in the absence of macrostructural brain injury. These changes may in turn be associated with life-long cognitive and behavioral consequences.^3–10,51^ Several other perinatal risk factors, including sex, focal brain injury, and SGA at birth, affect brain volumes in these vulnerable infants, with GA possibly the most prominent factor affecting both brain volumes and later outcome.^52^ In our study, the association between brain volumes and IGF-1 persisted when corrected for GA at birth and when infants with significant focal brain injury were removed or corrected for. In addition, and somewhat surprisingly, neither total energy intake nor SGA or extrauterine growth development, as measured by development in weight SDS from birth to time of MRI significantly contributed to brain volume at term. The influence of nutrition on the IGF-1-axis in extremely preterm infants has been suggested to mainly occur at 30–33 weeks PMA, thus after reaching a certain degree of maturity.^53^ Taken together, these results suggest that IGF-1 has an independent role in early postnatal brain development that could not be explained by general body growth or focal brain injuries.

An interesting finding in our study was that the most immature infants had the lowest serum levels of IGF-1, with a less pronounced increase with advancing postnatal age, and lacked the association with brain volumes. A possible explanation is that IGF-1 levels above a threshold value may be needed to promote brain development. A prolonged period with serum IGF-1 levels below 30 ng/ml has been related to retinal neurovascular morbidity in the preterm infant.^54^ A link between the angiogenic function of VEGF and IGF-1 has been shown, where IGF-1 acts as a permissive factor for VEGF in the neonate.^55–57^ In our study, median IGF-1 levels were below this value at all time points in the most immature group. In addition, adverse clinical events occur more frequently in the most immature infants and may, together with immaturity itself, have a more prominent role in brain growth and maturation than low IGF-1. It is also possible that the small number of infants in the subanalysis prevented any differences from reaching statistical significance.

Despite previous studies associating BDNF as well as VEGF and PDGF to brain development and disease experimentally and in adults,^17–20^ we did not find any significant association between these growth factors and preterm brain volumes. BDNF serum levels correlated with brain volumes, but the association did not remain after adjustment for immaturity and PMA at MRI. The lack of association could be due to the more dynamic circadian pattern of BDNF compared to IGF-1^58^ or the strong association of BDNF with GA.^59^ Another possible explanation for the associations with IGF-1, but not with BDNF, PDGF, and VEGF, might be due to the well-known, pronounced mitogenic role of circulating IGF-1 during the perinatal period extremely preterm infant. It is important to keep in mind that the brain-specific effect of these factors on a cellular level might be orchestrated and conducted via other influencing factors and mechanisms. The AUC in this study, calculated from circulating serum levels of BDNF, VEGF, and PDGF at PNDs 1, 7, 14, and 28, may thus not reflect the exact brain-specific action in extremely preterm infants during this particular phase of development. Furthermore, as recently described by Hellgren et al.,^25^ the endogenic longitudinal postnatal serum patterns of BDNF, VEGF, and PDGF do not follow the same postnatal pattern as IGF-1. IGF-1 is an agent in the somatotropic axis, and it is, as previously described, mainly produced in the liver and released into the bloodstream, whereas levels of BDNF, VEGF, and PDGF are likely more tightly linked to other circulating factors. For example, there are tight associations between circulating levels of BDNF, VEGF, and PDGF and platelet function^25^, which is linked to several clinical conditions such as sepsis and oxygen exposure. BDNF, which binds to different receptors, including TrK-B, has been of high interest during the last decades due to its essential roles in neuronal and synaptic properties, especially during the fetal developmental stages. BDNF activates several pathways, including the PI3-K/Akt, the PLC- γ, MAPK, and GTPases.^60^ Studies have found links between low levels of BDNF in newborns and autism spectrum disorder, and preterm infants with lower levels of BDNF had a higher probability of failing neurodevelopmental outcome tests.^61,62^ The roles of circulating VEGF, and PDGF in the preterm infant are not fully elucidated; however, they are both mediators of angiogenesis during development. In the preterm infant, potential links between VEGF and PDGF signaling, and dysregulation, inflammation, and altered vascular development have been suggested.^63,64^ VEGF is also a well-known mediator of neovascularization in the neurovascular disease ROP, which is associated with several outcomes such as brain volumes and poor neurodevelopmental outcome.^65^ It is important to further elucidate the complex interplay, function, and downstream mechanisms of circulating factors during different developmental phases in the extremely preterm.

### Limitations

MRI series not meeting the MRI quality volume acquisition criteria (Fig. 1), and a limited number of infants, especially in subanalysis, may have prevented us from detecting less pronounced associations. In addition, preterm infants constitute a heterogeneous group with numerous confounding factors associated with neonatal morbidity, and clinical interventions may have influenced brain development independent of IGF-1 levels.

## Conclusion

In conclusion, this study shows that higher circulating IGF-1 levels during the first four weeks of life are associated with increased total and regional brain volumes at TEA in extremely preterm infants. This effect was more pronounced in more mature infants with higher levels of IGF-1. Our findings suggest that IGF-1 promotes brain growth, which may protect the developing brain. The effects of systemically administered IGF-1/IGF-1BP3 on brain morphology and cognitive outcomes in extremely preterm infants are now being investigated in a randomized controlled trial (Clinicaltrials.gov: NCT03253263).

## Supplementary information

suppl_data/Supplement_Postnatal serum IGF-1 levels associate with brain volumes at term in extremely preterm infants

## Data Availability

Deidentified data analyzed in the current study are available from the corresponding author on reasonable request, in accordance with the jurisdiction of personal integrity.
