# Supplementary material for: Postnatal serum IGF-1 levels associate with brain volumes at term in extremely preterm infants
Source: Pediatr Res. 2022 Jun 9;93(3):666–74. doi: 10.1038/s41390-022-02134-4 (PMC9988684; doi:10.1038/s41390-022-02134-4)
Supplement: Supplementary file 1 — suppl_data/Supplement_Postnatal serum IGF-1 levels associate with brain volumes at term in extremely preterm infants [file 41390_2022_2134_MOESM1_ESM.pdf]

Supplement

Postnatal serum IGF-1 levels associate with brain volumes at term in extremely preterm infants

William Hellström MD[1], Lisa M. Hortensius MD[2-3], Chatarina Löfqvist PhD[4-5], Gunnel Hellgren PhD[4,6], Maria Luisa Tataranno MD PhD[2-3], David Ley MD PhD[7], Manon J.N.L. Benders MD PhD[2-3], Ann Hellström MD PhD[4], Isabella M. Björkman-Burtscher MD PhD[8], Rolf A. Heckemann MD PhD[9], Karin Sävman MD PhD[1,10]

[1]Department of Pediatrics, Institute of Clinical Sciences, Sahlgrenska Academy, University of Gothenburg, Gothenburg Sweden
[2] Department of Neonatology, Wilhelmina Children's Hospital, University Medical Center Utrecht, Utrecht University, Utrecht, The Netherlands
[3]University Medical Center Utrecht Brain Center, Utrecht University, Utrecht, The Netherlands
[4]Section for Ophthalmology, Department of Clinical Neuroscience, Institute of Neuroscience and Physiology, Sahlgrenska Academy, University of Gothenburg, Gothenburg, Sweden
[5]Institute of Health Care Science, Sahlgrenska Academy, University of Gothenburg, Gothenburg, Sweden
[6]Institute of Biomedicine, Sahlgrenska Academy, University of Gothenburg, Gothenburg, Sweden
[7] Department of Pediatrics, Institute of Clinical Sciences, Skåne University Hospital Lund, Lund, Skåne, Sweden
[8]Department of Radiology, Institute of Clinical Sciences, Sahlgrenska Academy, University of Gothenburg and Sahlgrenska University Hospital, Gothenburg, Sweden
[9]Department of Medical Radiation Sciences, Clinical Sciences, Sahlgrenska Academy, University of Gothenburg, Gothenburg, Sweden
[10]Region Västra Götaland, Department of Neonatology, The Queen Silvia Children's Hospital, Sahlgrenska University Hospital, Gothenburg, Sweden

Table of contents

Table of contents 2

Supplement Table 1. Dropout analysis 3

Supplement Table 2. Univariate analysis in initial statistical model 4

Supplement Table 3. Correlation of IGF-1 AUC PND 1-28 and all anatomical structures at TEA MR 5

Supplement Table 4. Relative regional brain volume distribution at TEA 8

Supplement Figure 1 Relative regional brain volume and IGF-1 AUC 9

Supplement Table 1. Dropout analysis

*Infants not included in final statistical analysis n=28**		***p*-value****
**Gestational age, median (range) weeks**	25.6 (22.7-27.9)	.74
**Birth weight, median (range) grams**	835 (415-1260)	.27
**Birth weight SDS, median (range)**	-0.24 (-5.2-0.8)	.07
**Total energy intake, median (range) kcal/kg/day*****	110.5 (61.3-136.8)	0.41
**Gender male, number (%)**	16 (57.1)	.73
**Sepsis, number (%)**	10 (35.7)	0.79
**NEC, number (%)**	1 (3.6)	1.0
**Any IVH or PVHI number (%)**	14 (50.0)	.19
**IVH Grade I-II, number (%)**	8 (28.6)	1.0
**IVH Grade III, number (%)**	2 (7.1)	.55
**PVHI, number (%)**	4 (14.3)	.18
**BPD, number (%)**	11 (39.3)	.19
**Any ROP, number (%)**	20 (71.4)	.30
**Time of MR, median (range) weeks**	NA	NA

Infants surviving to TEA MRI

****Compared to infants included in study analysis (n=49)***

*****one infant with incomplete data (excluded)***

Abbreviations. *SDS: standard deviation score, NEC: necrotizing enterocolitis, IVH: intraventricular hemorrhage, PVHI: periventricular hemorrhagic infarction, MRI: magnetic resonance imaging, ROP: retinopathy of prematurity, BPD: bronchopulmonary dysplasia, NA: not applicable, TEA: term equivalent age*

Supplement Table 2. Univariate analysis in initial statistical model

		**Un-standardized**		**Standardized**	
Linear regression	*p-value*	*B*	*Std. error*	*Beta*	*t*
**IGF-1 AUC PND 1-28**	<.001	1.36	0.27	0.60	5.13
**PMA at time of MR**	<.001	15.16	2.60	0.65	5.80
**GA in weeks**	<.01	15.08	4.78	0.42	3.15
**Weight SDS development****	.31	8.03	7.82	0.15	1.03
**Total energy intake PND 1-28**	.49	0.02	0.02	0.11	0.77
Mann Whitney U Test	*p-value*				
**Significant brain injury**	.05				
**Sepsis**	.44				
**Antenatal steroid treatment**	.79				
**SGA at birth**	.64				
**Sex**	.76				
**Treatment group*****	.88				

With total brain volume (cm3) as target variable, *********From birth until time of MRI, ***In randomized clinical trial #NCT 02760472*

Abbreviations: *IGF-1: insulin-like growth factor-1, AUC: area under the curve, PND: postnatal day, PMA: postmenstrual age, MRI: magnetic resonance imaging, GA: gestational age, SDS: standard deviation score, SGA: small for gestational age*

Supplement Table 3. Correlation of IGF-1 AUC PND 1-28 and all anatomical structures at TEA MR

**Brain region**	**Correlation**	***p*-value (unadjusted)**
White matter		
Insula WM R	0.58**	<.001[a]
Insula WM L	0.57**	<.001[a]
Parietal lobe WM R	0.50**	<.001[a]
Cingulate gyrus. ant WM R	0.48**	<.001[a]
Frontal lobe WM L	0.48**	.001[a]
Cingulate gyrus. post WM R	0.47**	.001[a]
Parietal lobe WM L	0.47**	.001[a]
Frontal lobe WM R	0.44**	.001
Ant temp lobe med WM L	0.44**	.002
Ant temp lobe med WM R	0.41**	.003
Sup temp gyrus middle WM R	0.41**	.004
Sup temp gyrus middle WM L	0.40**	.004
Cingulate gyrus. post WM L	0.39**	.006
G parahippocamp/amb ant WM L	0.38**	.008
Corpus Callosum m	0.36*	.01
Occipital lobe WM L	0.36*	.01
Ant temp lobe lat WM L	0.34*	.02
G parahippocamp/amb post WM L	0.34*	.02
Fusiform g post WM R	0.32*	.02
G parahippocamp/amb post WM R	0.32*	.03
Ant temp lobe lat WM R	0.32*	.03
G parahippocamp/amb ant WM R	0.32*	.03
Middle & inf temp gg ant WM L	0.32*	.03
Occipital lobe WM R	0.31*	.03
Fusiform g ant WM L	0.30*	.04
Sup temp gyrus post WM R	0.29*	.04
Fusiform g ant WM R	0.29*	.05
Fusiform g post WM L	0.25	.09
Middle & inf temp gg ant WM R	0.24	.10
Sup temp gyrus post WM L	0.19	.20
Middle & inf temp gg post WM R	0.15	.30
Cingulate gyrus. ant WM L	0.14	.33
Middle & inf temp gg post WM L	0.09	.54
Cortical gray matter		
Insula GM R	0.55**	<.001[a]

Insula GM L	0.49**	<.001[a]
Sup temp gyrus middle GM R	0.44**	.002
Frontal lobe GM L	0.43**	.002
Frontal lobe GM R	0.43**	.002
Sup temp gyrus post GM R	0.43**	.002
Sup temp gyrus middle GM L	0.41**	.003
Cingulate gyrus. ant GM R	0.39**	.005
Parietal lobe GM L	0.39**	.006
Sup temp gyrus post GM L	0.38**	.006
Parietal lobe GM R	0.38**	.007
G parahippocamp/amb post GM R	0.36*	.01
Cingulate gyrus. post GM L	0.35*	.01
Ant temp lobe lat GM R	0.34*	.02
Ant temp lobe lat GM L	0.34*	.02
Occipital lobe GM R	0.34*	.02
Cingulate gyrus. post GM R	0.34*	.02
G parahippocamp/amb post GM L	0.33*	.02
Occipital lobe GM L	0.32*	.02
Middle & inf temp gg ant GM R	0.28	.06
Middle & inf temp gg post GM R	0.27	.06
G parahippocamp/amb ant GM R	0.24	.09
Cingulate gyrus. ant GM L	0.24	.10
Fusiform g ant GM R	0.20	.18
Ant temp lobe med GM L	0.19	.19
Fusiform g post GM L	0.19	.20
Ant temp lobe med GM R	0.19	.20
Fusiform g post GM R	0.18	.23
Middle & inf temp gg ant GM L	0.18	.23
Middle & inf temp gg post GM L	0.17	.23
G parahippocamp/amb ant GM L	0.07	.65
Fusiform g ant GM L	-0.06	.69
Cerebellum		
Cerebellum L	0.47**	.001
Cerebellum R	0.47**	.001
Amygdala and hippocampus		
Hippocampus L	0.25	.08
Hippocampus R	0.22	.13
Amygdala R	0.20	.16
Amygdala L	0.14	.35
Deep gray matter		

Thalamus high int R	0.54**	<.001[a]
Lentiform Nucleus L	0.45**	.001
Lentiform Nucleus R	0.45**	.001
Thalamus high int L	0.44**	.002
Caudate nucleus R	0.32*	.03
Caudate nucleus L	0.25	.08
Thalamus low int L	0.13	.38
Thalamus low int R	0.10	.49
Subthalamic nucleus R	0.08	.59
Subthalamic nucleus L	0.05	.76
Brainstem		
Brainstem	0.39**	.005
Ventricles		
Lateral Ventricle R	-0.13	.37
Lateral Ventricle L	-0.06	.69

Abbreviations: IGF: insulin-like growth factor, AUC: area under the curve, PND: postnatal day, *TEA: term equivalent age, MR: magnetic resonance scanning, R: right, L: left, ant: anterior, post: posterior, sup: superior, inf: inferior, parahippocamp: parahippocampalis, amb: ambiens, g: gyrus, temp: temporal, lat: lateral, int: intensity. *indicating p<.05, **indicating p<.01* [a]*remained significant after adjusting for multiple testing according to Holm-Bonferroni*[1]

[1]Holm S. A Simple Sequentially Rejective Multiple Test Procedure. *Scandinavian Journal of Statistics*. 1979;6(2):65-70

Supplement Table 4. Relative regional brain volume distribution at TEA

Percentage of intracranial volume. Dichotomized infants with an IGF-1 AUC below median (IGF-1$_{low}$ n=25) and above median (IGF-1$_{high}$ n=24).

	Total n=49		**IGF-1$_{low}$ n=25**		**IGF-1$_{high}$ n=24**		
	Median	***Min-max***	***Median***	***Min-max***	***Median***	***Min-max***	***p-value***
White matter	***29.5***	24.4-35.0	29.5	25.7-34.7	29.1	24.4 35.0	.78
Cortical gray matter	***33.0***	24.3-38.3	33.0	24.3-38.0	32.9	28.8-38.3	.75
Deep gray matter	***4.4***	3.9-6.0	4.5	4.1-6.0	4.4	3.9-5.3	.41
Cerebellum	***5.5***	4.2-7.4	5.4	4.2-7.2	5.8	4.5-7.4	.02*

indicates p<.05. When adjusted for multiple testing according to Holm-Bonferroni, the association did not remain

Abbreviations: *IGF: insulin-like growth factor, n: number, min: minimum, max: maximum.*

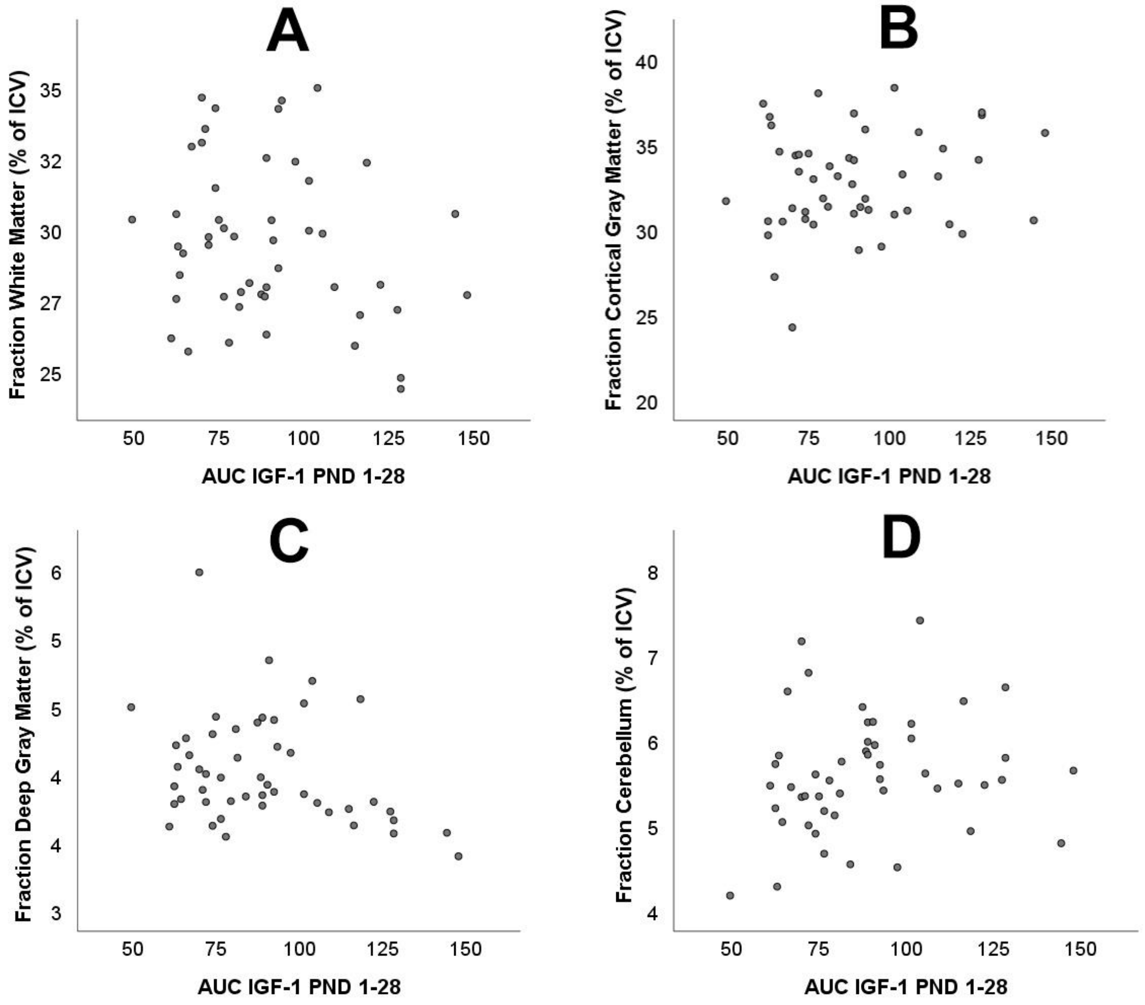

Supplement Figure 1 Relative regional brain volume and IGF-1 AUC

Scatter plots illustrating the distribution of relative regional brain volumes (adjusted for intracranial volume times 100) and IGF-1 AUC PND 1-28 in extremely preterm infants.

Abbreviations: IGF: insulin-like growth factor, AUC: area under the curve, PND: postnatal day
